# Haematological and Biochemical Parameters of Captive Siberian Tigers (*Panthera tigris altaica*) from the Heilongjiang Province, China

**DOI:** 10.1002/vms3.395

**Published:** 2020-12-13

**Authors:** Enqi Liu, Liying Ma, Dan You, Cen Yang, Yawen Hu, Haitao Xu, Dan Liu, Yajun Wang

**Affiliations:** ^1^ College of Wildlife and Protected Area Northeast Forestry University Harbin P. R. China; ^2^ Siberian Tiger Park Harbin P. R. China

**Keywords:** biochemical parameters, captive, haematological parameters, Siberian tigers

## Abstract

Haematological and biochemical parameters play important roles in safeguarding animal health and preventing disease, but the blood reference values of many wild animals are still unknown. Recently, few descriptions of the blood parameters of Siberian tigers (*Panthera tigris altaica*) have been reported because these tigers comprise an endangered species; however, it is extremely difficult to obtain blood samples necessary for these analyses. This study presents 14 haematological and 16 biochemical parameters of 133 Siberian tigers, of which 112 and 21 were from Heilongjiang Siberian Tiger Park (HB) and Hailin Siberian Tiger Park (HD), China, respectively. Our study is the first to determine the following parameters in Siberian tigers: red blood cell volume distribution width, platelet count, mean platelet volume, amylase (AMY), sodium/potassium, globulin and albumin/globulin levels. As the data for total bilirubin and AMY were not statistically significant, no statistical analysis was conducted for these parameters. Few parameters were significantly different according to sex and region (*p* < 0.05). The concentration of alkaline phosphatase decreased with age, whereas the creatinine (CREA) increased with age. The CREA concentration of tigers raised in HB was much lower than that of tigers raised in HD. The data obtained in this study provide a reference for monitoring the health of wild and captive Siberian tigers and will add important information to the standards for haematological and biochemical parameters of wild felines.

## Graphical abstract

This study collected and analysed the haematological and biochemical parameters of 133 Siberian tigers; these data can provide a reference for monitoring the health of wild and captive Siberian tiger.

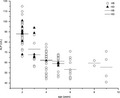

## 1 INTRODUCTION

The Siberian tiger (*Panthera tigris altaica*) is one of the largest felines, and is mainly found in Russian Far East, northeast of China and north of Korea. Currently, there are approximately 400 wild Siberian tigers globally, with only a few of these in China (Peng et al., 2020). Therefore, they are listed as level I protected animals by the Chinese government, are included in the Convention on International Trade in Endangered Species of Wild Fauna and Flora and are recognized as an endangered species by the International Union for Conservation of Nature (Goodrich et al., 2015; Liu et al., 2010).

The health of various animal organs can be evaluated using haematological and biochemical parameters. However, reports on the haematological and biochemical parameters of tigers are extremely limited, particularly those of Siberian tigers. Sajjad et al. (2012) and Farooq et al. (2012) determined the haematological and biochemical parameters of 10 Bengal tigers (*P. tigris tigris*) maintained in captivity at Lahore Zoo (*n* = 4) and in the seminatural environment of Lahore Wildlife Park (*n* = 6), Pakistan. They found that captivity has a significant effect on various haematological and biochemical parameters of these tigers. Larsson et al. (2015) assessed the haematological parameters of 16 captive Siberian tigers in the Zoological Park of the Sao Paulo Foundation. They noted higher leucocyte counts in male tigers, which may be partially explained by higher stress experienced by these individuals. They screened biochemical parameters of 10 of the 16 Siberian tigers and found greater albumin levels in male tigers, possibly related to the ingestion of larger quantities of meat (Larsson et al., 2017). Proverbio et al. (2020) conducted agarose gel electrophoresis of serum protein samples from 11 captive tigers: five Bengal tigers, five tigers (*P. tigris*) and one Siberian tiger. They found the electrophoretic patterns to be similar in all tigers. Moreover, except for the mean concentrations of α_2_ and β_1_ globulins, the mean values of albumin and globulin fractions for all tigers were within the range of reference values found in domestic cats. Sample sizes in the existing literature are extremely small to draw firm conclusions from, and these conclusions may lack generalizability.

This study aimed to further assess the baseline haematological and biochemical parameters of Siberian tigers. These values would be useful for monitoring the health of Siberian tigers and may be used as a reference for captive and free‐ranging Siberian tigers.

## 2 MATERIALS AND METHODS

### 2.1 Study area and experimental animals

We examined Siberian tigers (hereafter referred to as tigers) from two different Siberian Tiger Parks. Blood samples from 112 tigers (48 males and 64 females) from Heilongjiang Siberian Tiger Park (HB) and 21 tigers (7 males and 14 females) from Hailin Siberian Tiger Park (HD) were collected. All examined animals were 2–9 years old and weighed 98–442.4 kg. Their food consisted mainly of pork and chicken, and they were fasted for 1–2 days per week, with no physical or behavioural abnormalities observed before blood sample collection.

### 2.2 Sample collection

All tigers were fasted for 1 day before blood sample collection. Before examination, tigers were sedated within their own enclosures using anaesthetic darts containing ketamine (Jiangsu Zhongmu Beikang Pharmaceutical Co., Ltd., China) at 10 mg/kg. The animals were then weighed, and 5 ml of blood was collected aseptically from their jugular vein using disposable vacuum blood tubes without Na_2_ EDTA (Shanghai Liyin Precision Medical Products Co., Ltd., China). The sampled blood was placed at 20℃–25℃ for 30 min and then centrifuged at 489*g* for 10 min; then, 200‐μl supernatant (serum) was extracted from each sample for biochemical analysis. Following the same collection protocol, an additional 2 ml of whole blood was collected in vacuum blood tubes containing 2.7‐nM Na_2_ EDTA (Shanghai Liyin Precision Medical Products Co., Ltd., China) for haematological analysis. To minimize stress to the animals, collection procedures were standardized to the maximum extent possible. This included being performed by the same personnel, using the same techniques and at approximately the same time each day (between 9:00 a.m. and 12:00 a.m.).

### 2.3 Haematological analysis

Haematological parameters were assessed using an Exigo H400 automatic haematology analyzer, along with a specialized software (Tianliang Medical Equipment Co., Ltd., Taiwan), for analysing the haematological parameters of tigers. The haematological parameters evaluated were as follows:

White blood cell (WBC), Lymphocytes (LYM), Monocytes (MON), Neutrophils (NEU), Eosinophils (EOS), Red blood cell (RBC), Haematocrit (HCT), Red blood cell volume distribution width (RDW), Haemoglobin (HGB), Mean corpuscular volume (MCV), Mean corpuscular haemoglobin (MCH), Mean corpuscular haemoglobin concentration (MCHC), Platelet (PLT) and Mean platelet volume (MPV).

### 2.4 Biochemical analysis

Biochemical parameters were processed using the automatic veterinary biochemical analyzer Skyla VB1 with a specialized software (Tianliang Medical Equipment Co., Ltd., Taiwan), and for tiger blood biochemical analysis, 200‐μl serum was placed in Skyla VB1. The evaluated biochemical parameters are as follows:

Triglyceride (TG), Cholesterol (CHOL), Glucose (GLU), Total protein (TP), Albumin (ALB), Globulin (GLOB), Albumin/Globulin (A/G), Alkaline phosphatase (ALP), Alanine transaminase (ALT), Ureophil (UREA), Creatinine (CREA), Calcium (Ca), Phosphate (PHOS), Sodium (Na), Potassium (K), Sodium/Potassium (Na/K), Total bilirubin (TBIL) and Amylase (AMY).

### 2.5 Statistical analysis

Data were expressed as minimum, maximum, mean and standard deviation. The sex and area variables were analysed for normality using the Shapiro–Wilk normality test. Normally distributed data were analysed using an independent‐sample *t* test, and non‐normally distributed data were analysed using the Mann–Whitney *U*‐test. Statistical analyses were performed using IBM SPSS Statistics Software version 26. A value of *p* < 0.05 was considered statistically significant. As only two samples were obtained from 8‐year‐old tigers, the effect of age on the parameters could not be statistically analysed.

## 3 RESULTS

Currently, there are no globally acceptable reference ranges for all haematological and biochemical parameters of Siberian tigers. Except for the reference ranges of TG, CHOL, GLOB, A/G and Na/K levels, those of other haematological and biochemical parameters in this study were assessed using the specialized software developed by Tianliang Medical Equipment Co., Ltd., Taiwan. The two Siberian tiger parks have been using the specialized software to monitor whether the data are within the reference range provided by it, but the basis for establishing the reference range has not been made public. Although this reference range is not a standard data range for the genus, it provided a baseline reference to compare our results against. TBIL values were all lower than 6.8 μmol/L, while AMY values were between 1910 and 3,000 U L^−1^. As the TBIL and AMY data were not statistically significant, they have not been listed in the included tables.

The results of haematological and biochemical parameters of Siberian tigers confined to captivity at HB revealed that male tigers had significantly (*p* < 0.05) higher WBC, NEU, RBC counts, MCHC, TG, CHOL, PHOS and Na levels and significantly lower MCV, PLT count, MPV and A/G than female tigers. All other parameters were not significantly different between female and male tigers (Tables 1 and 2). The haematological parameters of tigers in the HD group did not significantly differ with sex (Table 3). Regarding the biochemical results, male tigers had significantly (*p* < 0.05) higher CHOL and PHOS levels and significantly lower GLU levels than female tigers (Table 4). Compared with tigers from HB, tigers from HD had lower HCT level, MCV, PLT count, MPV and ALT levels and higher LYM and MON count; MCHC, TP, ALB, UREA, CREA, Ca, PHOS, Na and K levels. There were no statistically significant differences in other parameters (Tables 5 and 6).

**TABLE 1:** Haematological values (Mean ± *SD*) of Siberian tigers confined to captivity at Heilongjiang Siberian Tiger Park

Haematology	Male (*n* = 48)		Female (*n* = 64)	
	Range	Mean ± *SD*	Range	Mean ± *SD*
WBC (4.50–26.40 × 10⁹/L)*	8.60–19.70	12.94 ± 2.78	5.70–26.30	10.99 ± 3.17
LYM (0.10–9.40 × 10⁹/L)	0.70–8.00	1.94 ± 1.17	0.80–3.90	1.70 ± 0.63
MON (0–2.40 × 10⁹/L)	0.10–1.00	0.28 ± 0.24	0.10–0.70	0.26 ± 0.18
NEU (0–7.70 × 10⁹/L)*	6.60–16.90	10.60 ± 2.51	4.70–22.90	8.90 ± 3.02
EOS (0–1.20 × 10⁹/L)	0.00–0.60	0.05 ± 0.12	0.00–0.40	0.04 ± 0.08
RBC (3.60–10.10 × 10^12^/L)*	5.82–11.05	7.33 ± 0.89	5.35–9.79	6.98 ± 0.78
HCT (21.00%–54.00%)	30.40–51.70	37.61 ± 3.85	28.90–49.70	36.73 ± 3.61
RDW (12.00%–17.50%)	14.60–17.20	15.70 ± 0.61	15.10–17.20	15.89 ± 0.47
HGB (6.30–18.70 g/dl)	12.60–24.10	15.79 ± 1.91	11.60–21.60	15.18 ± 1.67
MCV (33.60–92.30 fl)*	46.60–56.30	51.44 ± 2.17	47.30–57.60	52.74 ± 2.30
MCH (7.80–30.20 pg)	19.70–23.30	21.56 ± 0.68	20.20–23.10	21.77 ± 0.76
MCHC (24.10–50.00 g/dl)*	39.90–46.70	41.97 ± 1.12	39.40–43.60	41.31 ± 0.93
PLT (101–987 × 10⁹/L)*	63.00–278.00	191.29 ± 59.18	125.00–347.00	225.84 ± 46.85
MPV (5.50–10.50 fl)*	6.20–8.20	7.48 ± 0.38	7.00–8.80	7.79 ± 0.45

* Significant differences on haematological values between different sex tigers (*p* < 0.05).

**TABLE 2:** Biochemical values (Mean ± *SD*) of Siberian tigers confined to captivity at Heilongjiang Siberian Tiger Park

Biochemistry	Male (*n* = 48)		Female (*n* = 64)	
	Range	Mean ± *SD*	Range	Mean ± *SD*
TG (mmol/L)*	0.25–0.65	0.40 ± 0.11	0.15–0.57	0.32 ± 0.11
CHOL (mmol/L)*	3.95–9.87	5.91 ± 1.63	2.91–8.72	5.03 ± 1.15
GLU (0–19.70 mmol/L)	3.40–14.50	9.61 ± 2.52	6.30–15.00	9.91 ± 2.11
TP (45.00–87.00 g/L)	68.00–84.00	75.40 ± 4.32	66.00–86.00	74.45 ± 4.36
ALB (25.00–51.00 g/L)	30.00–37.00	34.10 ± 1.56	31.00–37.00	34.47 ± 1.40
GLOB (25.40–58.6 g/L)	35.00–52.00	41.29 ± 4.57	30.00–54.00	39.98 ± 4.73
A/G (0.70–1.30)*	0.60–1.00	0.84 ± 0.11	0.60–1.20	0.89 ± 0.12
ALP (8.00−483 U/L)	41.00–115.00	68.23 ± 18.15	44.00–97.00	70.59 ± 14.98
ALT (13.00−118 U/L)	42.00–121.00	61.33 ± 15.69	37.00–158.00	65.83 ± 22.63
UREA (3.90–17.9 mmol/L)	5.50–27.70	8.90 ± 4.27	6.10–21.10	8.36 ± 2.10
CREA (27.00−380 μmol/L)	168.00–734.00	255.27 ± 105.11	133.00–460.00	231.89 ± 54.20
Ca (2.10–3.30 mmol/L)	2.45–2.90	2.64 ± 0.10	2.40–2.93	2.62 ± 0.11
PHOS (1.13–3.65 mmol/L)*	1.00–2.23	1.64 ± 0.24	0.90–1.94	1.48 ± 0.24
Na (134−165 mmol/L)*	143.00–157.00	150.01 ± 2.93	143.00–154.00	148.91 ± 2.53
K (3.50–7.60 mmol/L)	4.20–5.50	4.98 ± 0.31	4.20–6.00	4.93 ± 0.35
Na/K	28.00–36.00	30.23 ± 1.87	26.00–35.00	30.41 ± 1.93

Note

For parameters of TG and CHOL, Tigers (*n* = 69), Males (*n* = 26) and Females (*n* = 43); the reference ranges of GLOB and A/G come from Proverbio et al. (2020).

* Significant differences on biochemical values between different sex tigers (*p* < 0.05).

**TABLE 3:** Haematological values (Mean ± *SD*) of Siberian tigers confined to captivity at Hailin Siberian Tiger Park

Haematology	Male (*n* = 7)		Female (*n* = 14)	
	Range	Mean ± *SD*	Range	Mean ± *SD*
WBC (4.50–26.40 × 10⁹/L)	7.50–20.40	14.32 ± 3.91	8.90–16.60	12.01 ± 2.34
LYM (0.10–9.40 × 10⁹/L)	1.40–5.70	3.54 ± 1.46	1.70–6.10	2.85 ± 1.07
MON (0–2.40 × 10⁹/L)	0.10–0.90	0.51 ± 0.28	0.20–1.10	0.35 ± 0.27
NEU (0–7.70 × 10⁹/L)	5.90–16.10	10.11 ± 3.18	5.60–10.80	8.62 ± 1.48
EOS (0–1.20 × 10⁹/L)	0.00–0.30	0.09 ± 0.12	0.00–0.90	0.11 ± 0.24
RBC (3.60–10.10 × 10^12^/L)	3.70–8.92	6.90 ± 1.59	6.25–8.83	7.13 ± 0.70
HCT (21.00%–54.00%)	21.20–39.00	35.11 ± 6.25	31.50–40.90	35.02 ± 2.50
RDW (12.00%–17.50%)	15.00–16.20	15.60 ± 0.42	14.90–17.00	16.01 ± 0.58
HGB (6.30–18.70 g/dl)	8.30–17.20	14.81 ± 2.96	13.60–18.30	15.10 ± 1.23
MCV (33.60–92.30 fl)	43.70–57.20	51.60 ± 4.29	46.00–54.30	49.29 ± 2.87
MCH (7.80–30.20 pg)	19.30–22.80	21.64 ± 1.23	20.10–22.50	21.24 ± 0.84
MCHC (24.10–50.00 g/dl)	39.20–44.20	42.04 ± 1.55	41.30–44.70	43.17 ± 1.23
PLT (101–987 × 10⁹/L)	87.00–197.00	129.86 ± 35.68	46.00–238.00	149.29 ± 53.94
MPV (5.50–10.50 fl)	6.50–8.00	7.27 ± 0.53	5.70–7.90	7.29 ± 0.53

**TABLE 4:** Biochemical values (Mean ± *SD*) of Siberian tigers confined to captivity at Hailin Siberian Tiger Park

Biochemistry	Male (*n* = 7)		Female (*n* = 14)	
	Range	Mean ± *SD*	Range	Mean ± *SD*
TG (mmol/L)	0.35–1.73	0.64 ± 0.49	0.24–0.78	0.39 ± 0.14
CHOL (mmol/L)*	3.84–6.73	5.47 ± 0.90	2.86–5.90	4.52 ± 0.77
GLU (0–19.70 mmol/L)*	6.50–9.60	8.11 ± 1.14	7.40–16.50	10.83 ± 2.59
TP (45.00–87.00 g/L)	76.00–81.00	77.71 ± 2.06	73.00–86.00	77.71 ± 3.27
ALB (25.00–51.00 g/L)	36.00–38.00	37.14 ± 0.69	34.00–38.00	36.43 ± 1.09
GLOB (25.40–58.6 g/L)	38.00–45.00	40.57 ± 2.64	38.00–50.00	41.86 ± 3.53
A/G (0.70–1.30)	0.80–1.00	0.93 ± 0.08	0.70–1.00	0.89 ± 0.07
ALP (8.00−483 U/L)	57.00–101.00	79.86 ± 18.04	60.00–95.00	74.50 ± 13.22
ALT (13.00−118 U/L)	35.00–54.00	43.57 ± 6.19	34.00–70.00	49.43 ± 11.41
UREA (3.90–17.9 mmol/L)	6.90–10.60	8.67 ± 1.32	6.40–13.10	9.19 ± 1.71
CREA (27.00−380 μmol/L)	611.00–935.00	766.71 ± 115.69	563.00–1158.00	811.43 ± 151.19
Ca (2.10–3.30 mmol/L)	2.43–2.90	2.71 ± 0.17	2.40–2.85	2.70 ± 0.13
PHOS (1.13–3.65 mmol/L)*	1.58–2.36	2.03 ± 0.31	1.32–2.00	1.71 ± 0.21
Na (134−165 mmol/L)	162.00–171.00	164.86 ± 3.67	152.00–168.00	161.86 ± 4.62
K (3.50–7.60 mmol/L)	4.80–6.40	5.67 ± 0.48	4.90–6.40	5.51 ± 0.48
Na/K	27.00–34.00	29.14 ± 2.27	25.00–33.00	29.57 ± 2.47

Note

The reference ranges of GLOB and A/G come from Proverbio et al. (2020).

* Significant differences on biochemical values between different sex tigers (*p* < 0.05).

**TABLE 5:** Haematological values (Mean ± *SD*) of Siberian tigers kept at Heilongjiang (HB) and Hailin (HD) Siberian Tiger Parks

Haematology	HB (*n* = 112)		HD (*n* = 21)	
	Range	Mean ± *SD*	Range	Mean ± *SD*
WBC (4.50–26.40 × 10⁹/L)	5.70–26.30	11.82 ± 3.15	7.50–20.40	12.78 ± 3.07
LYM (0.10–9.40 × 10/L)*	0.70–8.00	1.80 ± 0.91	1.40–6.10	3.08 ± 1.22
MON (0–2.40 × 10⁹/L)*	0.10–1.00	0.27 ± 0.20	0.10–1.10	0.40 ± 0.28
NEU (0–7.70 × 10⁹/L)	4.70–22.90	9.63 ± 2.93	5.60–16.10	9.12 ± 2.23
EOS (0–1.20 × 10⁹/L)	0.00–0.60	0.05 ± 0.10	0.00–0.90	0.10 ± 0.20
RBC (3.60–10.10 × 10^12^/L)	5.35–11.05	7.13 ± 0.84	3.70–8.92	7.05 ± 1.05
HCT (21.00%–54.00%)*	28.90–51.70	37.10 ± 3.72	21.20–40.90	35.05 ± 3.97
RDW (12.00%–17.50%)	14.60–17.20	15.81 ± 0.54	14.90–17.00	15.88 ± 0.56
HGB (6.30–18.70 g/dl)	11.60–24.10	15.44 ± 1.79	8.30–18.30	15.00 ± 1.91
MCV (33.60–92.30 fl)*	46.60–57.60	52.19 ± 2.33	43.70–57.20	50.06 ± 3.48
MCH (7.80–30.20 pg)	19.70–23.30	21.68 ± 0.73	19.30–22.80	21.37 ± 0.98
MCHC (24.10–50.00 g/dl)*	39.40–46.70	41.59 ± 1.06	39.20–44.70	42.80 ± 1.41
PLT (101–987 × 10⁹/L)*	63.00–347.00	211.04 ± 54.99	46.00–238.00	142.81 ± 48.59
MPV (5.50–10.50 fl)*	6.20–8.80	7.65 ± 0.45	5.70–8.00	7.29 ± 0.51

* Significant differences on haematological values between different sex tigers (*p* < 0.05).

**TABLE 6:** Biochemical values (Mean ± *SD*) of Siberian tigers kept at Heilongjiang (HB) and Hailin (HD) Siberian Tiger Parks

Biochemistry	HB (*n* = 112)		HD (*n* = 21)	
	Range	Mean ± *SD*	Range	Mean ± *SD*
TG (mmol/L)	0.15–0.65	0.35 ± 0.12	0.24–1.73	0.48 ± 0.31
CHOL (mmol/L)	2.91–9.87	5.36 ± 1.41	2.86–6.73	4.83 ± 0.92
GLU (0–19.70 mmol/L)	3.40–15.00	9.78 ± 2.29	6.50–16.50	9.92 ± 2.54
TP (45.00–87.00 g/L)*	66.00–86.00	74.86 ± 4.35	73.00–86.00	77.71 ± 2.87
ALB (25.00–51.00 g/L)*	30.00–37.00	34.31 ± 1.48	34.00–38.00	36.67 ± 1.02
GLOB (25.40–58.6 g/L)	30.00–54.00	40.54 ± 4.69	38.00–50.00	41.43 ± 3.25
A/G (0.70–1.30)	0.60–1.20	0.87 ± 0.12	0.70–1.00	0.90 ± 0.07
ALP (8.00−483 U/L)	41.00–115.00	69.58 ± 16.38	57.00–101.00	76.29 ± 14.76
ALT (13.00−118 U/L)*	37.00–158.00	63.90 ± 20.00	34.00–70.00	47.48 ± 10.21
UREA (3.90–17.9 mmol/L)*	5.50–27.70	8.59 ± 3.21	6.40–13.10	9.02 ± 1.58
CREA (27.00−380 μmol/L)*	133.00–734.00	241.91 ± 80.50	563.00–1158.00	796.52 ± 139.07
Ca (2.10–3.30 mmol/L)*	2.40–2.93	2.63 ± 0.10	2.40–2.90	2.70 ± 0.14
PHOS (1.13–3.65 mmol/L)*	0.90–2.23	1.55 ± 0.25	1.32–2.36	1.82 ± 0.29
Na (134−165 mmol/L)*	143.00–157.00	149.38 ± 2.75	152.00–171.00	162.86 ± 4.48
K (3.50–7.60 mmol/L)*	4.20–6.00	4.95 ± 0.33	4.80–6.40	5.56 ± 0.47
Na/K	26.00–36.00	30.33 ± 1.90	25.00–34.00	29.43 ± 2.36

Note

For parameters of TG and CHOL, Tigers (*n* = 90), HB (*n* = 69) and HD (*n* = 21); the reference ranges of GLOB and A/G come from Proverbio et al. (2020).

* Significant differences on biochemical values between different sex tigers (*p* < 0.05).

**FIGURE 1:**
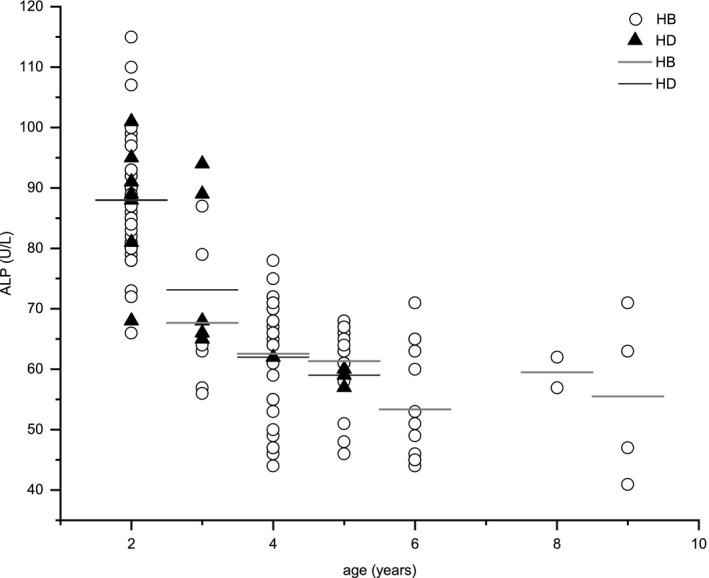
The relationship between the mean concentration of alkaline phosphatase (ALP) and age in Heilongjiang (HB) and Hailin (HD) Siberian Tiger Parks. *Bars* represent the average value of each dataset

**FIGURE 2:**
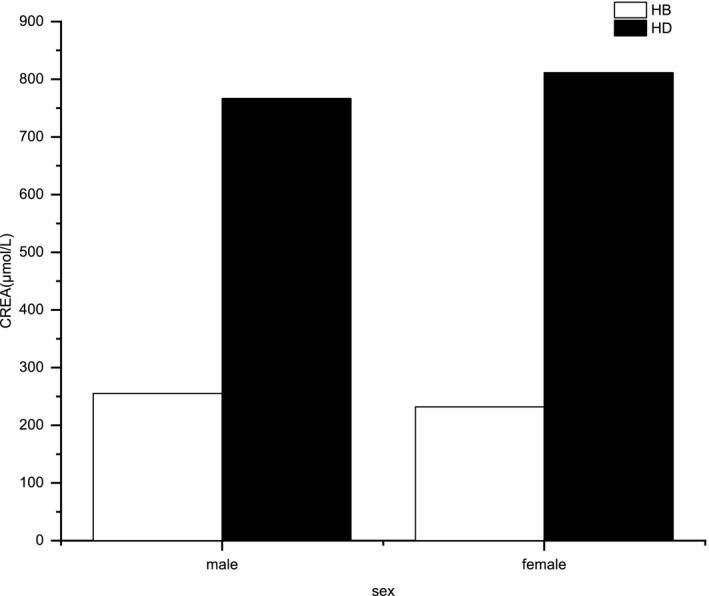
The relationship between the mean concentration of creatinine (CREA) and different sex in Heilongjiang (HB) and Hailin (HD) Siberian Tiger Parks

**FIGURE 3:**
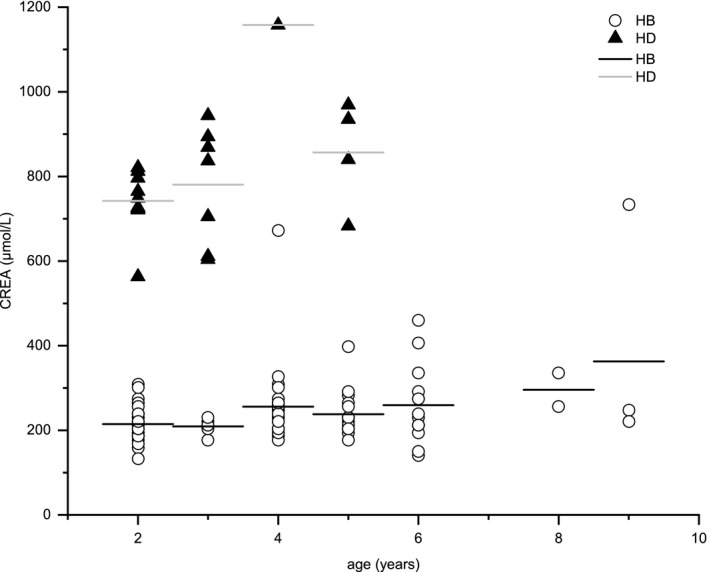
The relationship between the mean concentration of creatinine (CREA) and age in Heilongjiang (HB) and Hailin (HD) Siberian Tiger Parks. *Bars* represent the average value of each dataset

The differences in ALP and CREA levels were the most consistent among all the parameters. Figure 1 shows the relationship between ALP concentration and age in the two regions, indicating that ALP concentration decreased with age, except for two tigers: an 8‐ and a 9‐year‐old. Figures 2 and 3 show the relationships of CREA concentration with sex and age in the two populations. Figure 2 indicates that the CREA concentration of male and female tigers from HB was lower than that of tigers from HD. As shown in Figure 3, CREA level increased with age, especially in samples from 4‐year‐old tigers, and this level was extremely high in tigers from HD. No obvious linear relationships between other parameters and age, or sex were observed.

## 4 DISCUSSION

The present study enrolled 133 Siberian tigers maintained at two different Siberian Tiger Parks; our results will further improve the baseline haematological and biochemical parameters for Siberian tigers.

WBC, MON, NEU, RBC counts, HCT, HGB, MCH, MCV, GLU, TP, ALB, ALP, ALT, Na and K levels of the tigers examined in this study were consistent with those measured by Larsson et al. (2015) and Larsson et al. (2017). EOS count in our study was lower but the LYM value was higher than the above studies; these differences might be related to the excitement or ‘alarm reaction’ caused by ketamine in the anaesthesia used (Kocan et al., 1985). TG and CHOL levels were much lower, which might be related to the low activity level of captive tigers in their space‐limited enclosures. Nevertheless, the concentrations of MCHC, Ca and PHOS obtained in this study were much higher than those reported by Larsson et al. (2015, 2017). The probable reason is that most tigers aged from 2 to 5 years were fed additives containing iron, vitamin D_3_, Ca and PHOS to promote RBC count and HGB concentration (Larsson et al., 2015) as well as juvenile bone growth (Moen et al., 2010). NEU and RBC counts as well as HGB, ALT and Na levels were higher than corresponding reference ranges; which might be related to age, physiological condition, stress level, altitude and diseases, such as hypernatraemia, hyperthyroidism or hepatocellular damage (Larsson et al., 2017; Widmer et al., 2012).

Figures 1, 2, 3 show changes in the regularity of ALP and CREA levels. The ALP concentration of the younger tigers was higher than that of the older tigers. As ALP can hydrolyse phosphate and pyrophosphate during osteogenesis, promoting mineral formation and mineralization in bones, it is beneficial to bone formation (Jiang et al., 2019). ALP concentrations were increased in tigers over 8 years. However, the relevance of this finding is unknown, owing to the contingent small sample size and similar sex of the sampled animals (Figure 1). The CREA concentration of male and female tigers from HD was higher than that of tigers from HB, which may be due to the fact that the tigers maintained at HD consumed an additional kilogram of food per day than those maintained at HB, and their muscle mass was generally higher (Miller et al., 1999). Moreover, adult tigers consumed more meat; this could be one of the reasons for CREA level increased with age (Figure 3) (Beltrán et al., 1991). CREA concentrations in the 4‐year‐old tiger from HD were abnormally high, but as there was only one sample from this age, the result might be an anomaly.

As the main parameter of the renal function examination, UREA level in this study was extremely high, and the reason for this was similar to that for the high CREA level (Marco et al., 2000). Dehydration, acute haemorrhage and a series of kidney diseases, such as glomerulonephritis and renal failure, could increase CREA and UREA levels. In addition, TBIL is an important parameter of liver function and is the main factor for diagnosing jaundice. In this study, the concentrations of TBIL were all <6.8 μmol/L. Higher TBIL levels might lead to conditions such as jaundice, hepatitis and hepatonecrosis.

The RDW, PLT count, MPV, AMY level and Na/K of Siberian tigers were not investigated in previous studies. Although Proverbio et al. (2020) have screened GLOB level and A/G, these values were determined from only one Siberian tiger sample, thus lacking generalizability. Therefore, our study is the first to screen these parameters, broadening the baseline haematological and biochemical parameters of Siberian tigers.

RDW reflects the variations in the size of circulating RBCs. Increases in RDW are related to various conditions including anaemia caused by the deficiency of iron, vitamin B_12_ or folate; haemolysis caused by RBC destruction; malnutrition; liver disease; and cancer (Tseliou et al., 2014).

PLTs mainly play a haemostatic role in the body. In this study, some of the PLT counts were lower than the reference range. This could be attributed to the similar size of RBCs and PLTs and the aggregation of PLTs in vitro, owing to which the automatic haematology analyzer excluded few PLTs (Norman et al., 2001). Reduced PLT level is a common finding in idiopathic thrombocytopenic purpura, acute leukaemia and disseminated intravascular coagulation.

The main function of MPV is to identify the cause of thrombocytopenia (May et al., 2019). Under inflammatory conditions or in immune thrombocytopenic purpura or myeloproliferative diseases, the MPV increases, whereas in aplastic anaemia or hypersplenism, MPV decreases (Iida et al., 2018).

AMY is one of the markers most commonly used for diagnosing pancreatic diseases, especially acute pancreatitis (Vissers et al., 1999). In this study, AMY level ranged from 1910 to 3,000 U L^−1^ with an average value of 2,563.45 U L^−1^. The AMY level for most tigers was approximately 3,000 U L^−1^, which could be interpreted as a sign of disease but was most likely related to a high‐fat, high‐protein or high‐carbohydrate dietary (Grossman et al., 1943; Kondo et al., 1988).

Similar to Na and K, serum Na/K reflects the osmotic pressure and acid–base balance of the body and ensures normal functioning of cell and neuromuscular activity; however, there is currently limited data published on this topic. Thus, the Na/K values obtained in this study can provide a basis for future research in relevant fields.

GLOB plays an important role in immunity, in addition to being related to the liver. Therefore, changes in GLOB level are not only related to liver diseases but also to chronic inflammation and some types of tumours (such as multiple myeloma and lymphoma) (He et al., 2017). As a marker for systemic inflammation, low A/G indicates a systemic inflammatory response (Niwa et al., 2018).

## 5 CONCLUSION

Haematology is an important tool for assessing the physiological status of individuals and often provides the first and only indicator of a disease. Blood biochemical test is also one of the most important tests for initial diagnosis and treatment. However, many factors, including biological, methodological, dietary and management variables, influence the haematological and biochemical parameters of captive wild animals. The results of this study are similar to those reported previously; however, our study is the first to analyse the haematological and biochemical parameters in a large sample of Siberian tigers and the first to report certain specific parameters.

This new report aims to further improve our understanding of the haematological and biochemical parameters of Siberian tigers, and these values will be useful for monitoring the health of captive and free‐ranging Siberian tigers. Future work will involve the collection of more samples from captive and free‐ranging Siberian tigers from various provinces in China for the detection and analysis of other haematological and biochemical parameters.

## CONFLICT OF INTEREST

The authors declare that they have no conflict of interest.

## AUTHOR CONTRIBUTION

**Enqi Liu:** Writing‐original draft. **Dan You:** Formal analysis. **Liying Ma:** Data curation. **Cen Yang:** Investigation. **Yawen Hu:** Investigation. **Haitao Xu:** Resources. **Dan Liu:** Resources. **Yajun Wang:** Conceptualization; Funding acquisition; Project administration; Writing‐review & editing.

## ETHICAL APPROVAL

The authors confirm that the ethical policies of the journal, as noted on the journal's author guidelines page, have been adhered to and the appropriate ethical review committee approval has been received.

### Peer Review

The peer review history for this article is available at https://publons.com/publon/10.1002/vms3.395.
